# Isoorientin Improves Excisional Skin Wound Healing in Mice

**DOI:** 10.3390/ph17101368

**Published:** 2024-10-14

**Authors:** Aline B. Hora, Laiza S. Biano, Ana Carla S. Nascimento, Zaine T. Camargo, Greice I. Heiden, Ricardo L. C. Albulquerque-Júnior, Renata Grespan, Jessica M. D. A. Aragão, Enilton A. Camargo

**Affiliations:** 1 Graduate Program in Health Sciences, Federal University of Sergipe, São Cristóvão 49060-676, Brazil; 2 Graduate Program in Physiological Sciences, Federal University of Sergipe, São Cristóvão 49107-230, Brazil; 3 Graduate Program in Chemistry, Federal University of Sergipe, São Cristóvão 49107-230, Brazil; 4 Graduate Program in Dentistry, Federal University of Santa Catarina, Florianópolis 88040-900, Brazil

**Keywords:** flavone, tissue repair, collagen deposition, epidermal thickness

## Abstract

**Background/Objectives**: Wound healing relies on a coordinated process with the participation of different mediators. Natural products are a source of active compounds with healing potential. Isoorientin is a natural flavone recognized as having several pharmacological properties, such as anti-inflammatory effects, making it a potential treatment for wounds. We investigated the effect of isoorientin on the healing of excisional skin wounds. **Methods**: Male Swiss mice were subjected to the induction of excisional skin wounds (6 mm diameter) and treated with a vehicle (2% dimethyl sulfoxide in propylene glycol) or 2.5% isoorientin applied topically once a day for 14 days. The wound area was measured on days 0, 3, 7, and 14. Histopathological analyses were performed on the cicatricial tissue after 14 days. The myeloperoxidase activity and the interleukin-1β, tumoral necrosis factor (TNF)-α, and interleukin-6 concentrations were determined on the third day. **Results**: We observed that 3 days after the topical application of isoorientin, the lesion area was significantly smaller when compared to those of the vehicle (*p* < 0.01) and control (*p* < 0.05) groups. No difference was observed after 7 and 14 days of induction. Despite this, on day 14, histological analysis of cicatricial tissue from the animals treated with isoorientin showed reduced epidermal thickness (*p* < 0.001) and increased collagen deposition (*p* < 0.001). These effects were accompanied by decreased myeloperoxidase activity and interleukin-1β concentration on the third day of induction, without alteration in TNF-α and interleukin-6. **Conclusions**: The treatment with isoorientin promoted better tissue repair in excisional wounds in mice, which may be linked to the modulation of the early inflammatory response.

## 1. Introduction

Skin wounds have a significant impact on the health and quality of life of patients [1], not only due to the disruption of skin integrity and the primary outcomes of this situation, but also due to other factors such as sleep disturbances, fatigue, job loss, low self-esteem, and depression [2].

The main causes of skin wounds are physical, chemical, or thermal injuries that lead to the disruption of skin integrity. Many wounds present impaired healing, both acutely and chronically, as alterations can occur in the normal stages of healing, such as those caused by infectious conditions [3]. The healing of skin wounds is regulated by a standard succession of coordinated events, involving the phases of hemostasis, inflammation, proliferation, and remodeling [4].

Wound treatment involves numerous conventional resources that are generally used topically, such as essential fatty acids, alginates, antiseptics, activated charcoal, proteolytic enzymes, hydrocolloids, hydrogels, hydropolymers, silver sulfadiazine, growth factors, and skin substitutes, among others [5]. However, for most of these treatments, there is a need for greater accessibility to the population, as well as simpler and less costly production [6]. Furthermore, there are controversies about the safety and efficacy of wound treatment [7,8].

Thus, new approaches are needed to treat skin wounds, and natural products are a historically relevant alternative for treating various diseases [9,10]. Within the scope of natural products, flavonoids are polyphenolic compounds that have been widely studied for their anti-inflammatory and antioxidant properties [11,12]. According to previous studies, flavonoids have been mentioned as promising therapeutic agents for the healing of wounds [13], since they can modulate the nuclear factor (NF)-κB signaling pathway, which regulates the expression of inflammatory genes and is closely associated with chronic inflammation [12,14].

Isoorientin is a natural flavonoid that belongs to the flavones subclass. This compound is found in various plants, such as passion fruit, corn, wheat, and barley, and is used in traditional medicine in some cultures [15]. It has attracted attention due to its potential healing properties, which may be associated with various biological mechanisms. At the cellular level, it exerts anti-inflammatory effects by inhibiting pro-inflammatory mediators, such as the cytokines tumoral necrosis factor (TNF)-α, interleukin (IL)-1β, and IL-6 [16,17]. A previous study showed that Swiss 3T3 albino mouse fibroblast exposure to isoorientin improved in vitro wound closure by enhancing cell migration [18]. These authors suggested that isoorientin stimulated cellular nutrition and collagen synthesis, which is crucial for reconstructing damaged tissue. However, the potential of isoorientin for wound healing in vivo has not been previously evaluated. In the present study, we explored the effect of topically applied isoorientin on the healing of excisional wounds induced in mice. We showed for the first time that this isoflavone produces a beneficial effect on the wound healing process in mice, especially when linked with its anti-inflammatory action.

## 2. Results

### 2.1. Isoorientin Reduces the Wound Area during the Inflammatory Phase

**Figure 1:**
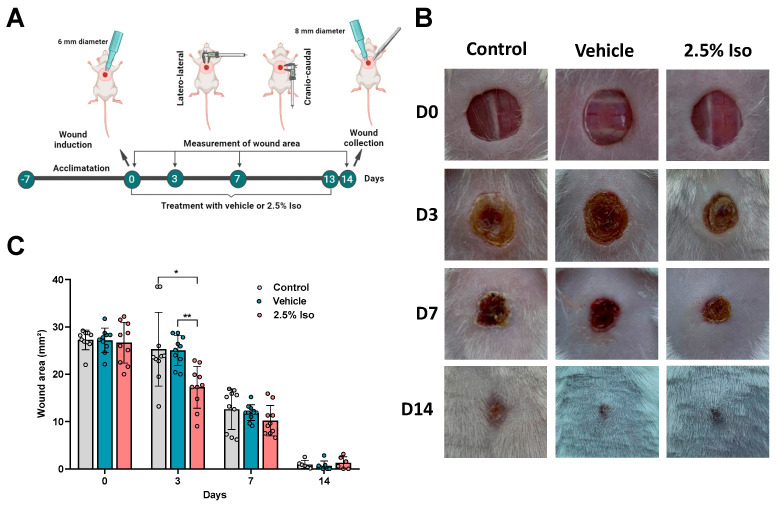
The effect of isoorientin on the skin wound area at different time points. Mice were divided into control (no treatment), vehicle (2% dimethyl sulfoxide (DMSO) in propylene glycol), and 2.5% isoorientin (2.5% Iso) groups. The area of each skin lesion was measured at days 0, 3, 7, and 14 after induction, according to the experimental design (**A**). Representative images of the skin lesions of the groups at different time points are shown (**B**). The area of the skin lesions (**C**) is expressed as mean ± SD (n = 10/group at D0, 3, and 7, and n = 6/group at D14). Two-way analysis of variance (ANOVA) and the Bonferroni post hoc test were used. * *p* < 0.05 or ** *p* < 0.01 compared to the control or vehicle group, as indicated. Created in BioRender.com.

Wounds were inflicted on day 0 and treated over 14 days, according to the experimental design (Figure 1A). The areas of the wounds were similar at day 0. However, the group treated with isoorientin (2.5% Iso) showed a smaller wound area on the third day, as observed in the representative images (Figure 1B) and the quantitative data (Figure 1C), compared to the vehicle (*p* < 0.01) and control (*p* < 0.05) groups. This parameter remained similar in all groups on the seventh and fourteenth days (Figure 1B,C).

### 2.2. The Administration of Isoorientin Enhances the Quality of Wound Healing

**Figure 2:**
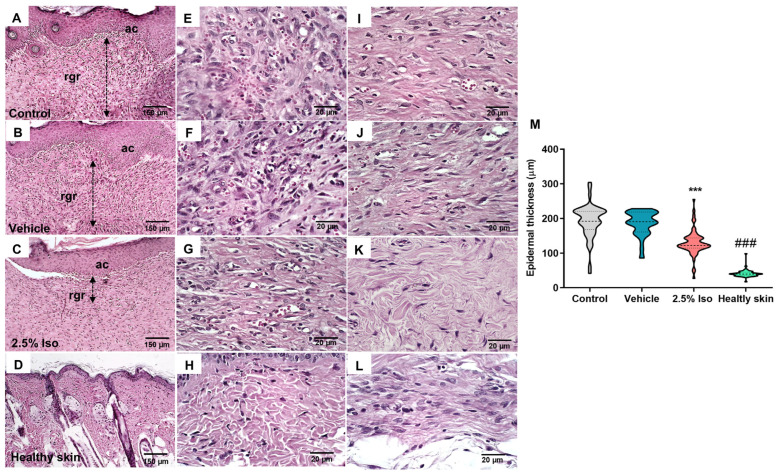
Representative images of the wound tissues 14 days after induction and quantification of the epidermal thickness. Histological sections stained with hematoxylin and eosin represent each experimental group’s epidermis and papillary/reticular dermis. Panoramic views of the groups—control (**A**), vehicle (**B**), 2.5% Iso (**C**), and healthy skin (**D**)—show the measurement of the granulation reaction depth (dotted lines with double arrows), epidermal acanthosis (ac), and residual granulation reaction (rgr) in the lamina propria (100×). Details of the granulation reaction and the deeper dermal portion in the control (**E**,**I**), vehicle (**F**,**J**), 2.5% Iso (**G**,**K**), and healthy skin (**H**,**L**) groups (400×) are shown. A violin plot shows the measurement of the mean epidermal thickness in the experimental groups (**M**), n = 6 animals with 6–8 measurements each. The Kruskal–Wallis and Dunn’s post hoc tests were used. *** *p* < 0.001 and ^###^ *p* < 0.001 compared to the control or vehicle groups.

The control and vehicle groups exhibited similar histopathological features after 14 days, with intense epidermal acanthosis occurring and the papillary/reticular dermis presenting relatively thick residual granulation tissue consisting of numerous capillary vessels with an irregular distribution, supported by collagen fibrils arranged in a reticular pattern (Figure 2A–F). Additionally, a thicker and deeper band of granulation tissue was observed in these groups compared to the narrow subepidermal band seen in the 2.5% Iso group (Figure 2G–I). The granulation tissue was highly vascularized in the control and vehicle groups and was more fibrocellular in the 2.5% Iso group, but was absent in normal skin (Figure 2).

The epidermal thicknesses in the control and vehicle groups were statistically similar (*p* > 0.05), but both were significantly thicker than in the 2.5% Iso group and healthy skin (*p* < 0.001, Figure 2M).

### 2.3. Isoorientin Promotes Greater Collagen Deposition in Injured Tissue

**Figure 3:**
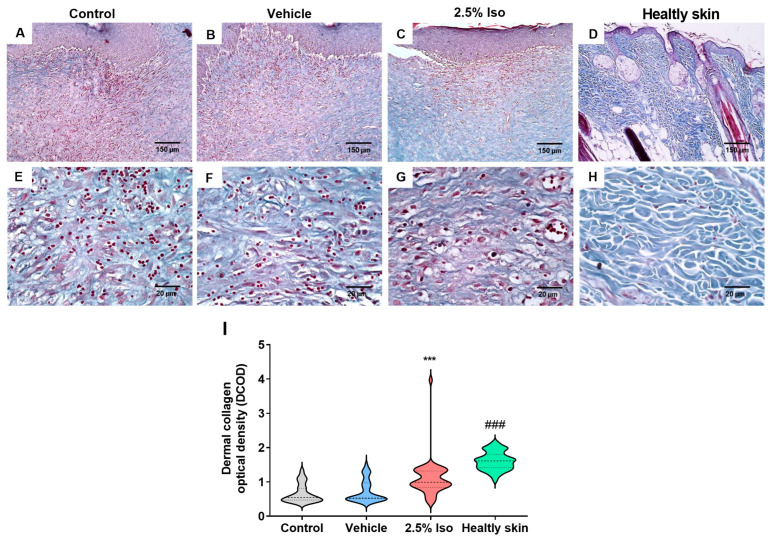
Representative images of Masson’s trichrome staining, and quantification of collagen deposition in the wound tissues 14 days after induction. Histological sections stained with Masson’s trichrome represent each experimental group’s papillary/reticular dermis area. Panoramic views of the control (**A**), vehicle (**B**), 2.5% Iso (**C**), and healthy tissue (**D**) groups (100×) are shown, along with the histological appearances of collagen fibers in the experimental groups and normal skin (**E**–**H**) (400×). The violin plot demonstrates the dermal collagen optical density (DCOD) determination in the experimental groups (**I**), n = 6 animals with 7–10 measurements each. Data are expressed as the median and minimum to maximum. Kruskal–Wallis and Dunn’s post hoc tests were used. *** *p* < 0.001 and ^###^ *p* < 0.001 compared to the control or vehicle groups.

The collagen fibers deposited in the dermis were identified by blue staining, using Masson’s trichrome stain (Figure 3A–H), after 14 days. The control and vehicle groups showed a similar pattern of scar collagenization, represented by thin bundles of varying lengths and irregular arrangement of collagen fibers. The 2.5% Iso group also exhibited thin, delicate fibers, but they were more densely packed and arranged in parallel patterns (Figure 3G). Healthy skin displayed a dense and compact collagenization, consisting of thick and coarse bundles of collagen fibers arranged in an intertwined pattern (Figure 3H).

The control and vehicle groups exhibited similar density values for the collagenization density. However, the 2.5% Iso group and healthy skin showed a higher density of collagen deposition compared to the control and vehicle groups (*p* < 0.001) (Figure 3I).

### 2.4. Inflammatory Markers Are Reduced in the Wounds of Animals Treated with Isoorientin

**Figure 4:**
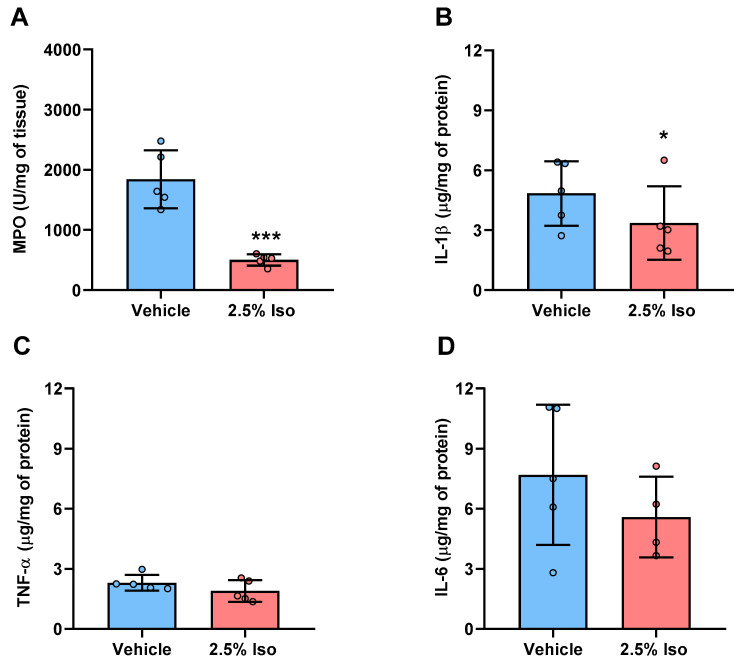
The effect of isoorientin on the myeloperoxidase activity and pro-inflammatory cytokine production in the wound tissues 3 days after induction. Mice were treated daily according to their respective groups: control (no treatment), vehicle (2% DMSO in propylene glycol), or Iso (2.5% Isoorientin). The wounds were analyzed on the third day to measure MPO activity (**A**) and the concentrations of IL-1β (**B**), TNF-α (**C**), and IL-6 (**D**). Data are expressed as mean ± SD (n = 5). Two-way ANOVA and the Bonferroni post hoc test were used. * *p* < 0.05 and *** *p* < 0.0001, respectively, compared to the vehicle group.

The activity of the myeloperoxidase (MPO) enzyme was evaluated on the third day, to indirectly determine the neutrophil infiltrate in the wounds. Since we did not observe differences between the healing parameters in the control and vehicle groups, only the vehicle group was used for comparison with the 2.5% Iso group. We found a reduction in MPO activity in the wounds of the 2.5% Iso group (*p* = 0.0003) compared to the vehicle group (Figure 4A).

Furthermore, the pro-inflammatory cytokines IL-1β, TNF-α, and IL-6 were measured in the wounds on the third day. Treatment with isoorientin reduced the amount of IL-1β (*p* = 0.029; Figure 4B) compared to the vehicle group. However, this effect was not observed for the TNF-α and IL-6 cytokines (Figure 4C,D).

## 3. Discussion

In this study, we showed for the first time that isoorientin exhibits wound-healing activity in vivo. Our data suggest that this effect might be linked to its anti-inflammatory activity.

For this, we used the excisional skin wound model in mice. The analysis was initially focused on wound closure by secondary intention, using previously established parameters [19]. The healing of these excisional wounds significantly differs from the process associated with incisional wounds. Due to their extent and the lack of approximation of the wound edges, the closure of excisional wounds relies on tissue contraction, requiring increased cell proliferation, granulation tissue formation, and extracellular matrix deposition in the affected area [19].

Natural products have long been used for wound treatment to improve healing [20]. Isoorientin has several pharmacological properties, including antioxidant and anti-inflammatory effects [21]. Based on these properties, isoorientin seems to be of interest for the healing process as it possibly acts through various biological mechanisms that facilitate tissue repair.

In this study, we observed a reduction in wound area in animals treated with isoorientin at a concentration of 2.5% during the inflammatory phase. The healing of these lesions can be impaired both in the short and long term due to potential disruptions in the normal stages of recovery, such as those caused by infections [22]. The gross features of the wounds also showed that the crust formed in the treated group was thinner, with less fibrin deposition used for its formation. By the third day after the injury, lesions from all groups were neither edematous nor presenting surrounding necrotic areas. Thus, the reduction in wound area during the inflammatory phase aided the repair over the closing time (14 days). The relevance of this finding is amplified by the knowledge that cutaneous lesions resulting from a diverse range of damages can have their recovery compromised by factors that alter the normal stages of healing, including exacerbated inflammation, which delays the recovery process [23,24,25]. Inflammation, as a natural response to injury, plays a crucial role in this context, involving a complex interplay of mediators that, if dysregulated, can lead to significant disturbances [3,22,26]. This emphasizes the importance of effective therapeutic approaches, as observed for isoorientin.

The impact of isoorientin’s action in the initial period of the wound healing process seemed to contribute to a better histological pattern of the cicatricial tissue. Although all groups achieved re-epithelialization on day 14 after wound induction, it is important to note that treatment with isoorientin promoted better reorganization of collagen fibers. Of interest is the fact that some flavonoids have been recognized by their wound-healing effects and their influence on collagen reorganization in diabetic animals [27], such as quercetin in mice [28] and a flavonoid-rich fraction from *Martynia annua* Linn or luteolin in rats [29].

The effectiveness of wound healing depends on the multifactorial phenomenon. It encompasses initial hemostasis, essential for containing blood loss and controlling inflammation, which precedes the repair stage [4]. The decrease in epidermal thickness and the increase in the density of collagen deposition observed after treatment with isoorientin suggest positive modulation in cell proliferation and migration, neovascularization, and epithelial growth, emphasizing the impact of this flavone on wound healing and dermal restructuring. Additionally, the wounds treated with isoorientin presented attenuated inflammatory infiltrate and a marked reduction in interstitial thickening. These data suggest that isoorientin favors collagen reorganization and wound healing by modulating the inflammatory response and tissue remodeling.

Skin lesion repair is characterized by angiogenesis, fibroplasia, and re-epithelialization, and it involves the migration and proliferation of many cells, including fibroblasts, endothelial cells, and epithelial cells; the deposition of connective tissue; and wound contraction [30]. It is known that the movement of macrophages and fibroblasts to the wound area is desirable for building complete functional tissue. Monocyte infiltration into the wound area, following the migration of polymorphonuclear leukocytes and the subsequent stimulation of macrophage differentiation, causes the secretion of key growth factors and cytokines [3].

Pro-inflammatory cytokines, such as IL1-β, IL-6, and TNF-α, play a central role in wound healing mechanisms [16]. Isoorientin reduced neutrophil migration (detected through the MPO activity) and the concentration of IL1-β in treated wounds after 3 days of induction, indicating that it can modulate the responses of cells involved in the initial stages of the inflammatory process, accelerating tissue repair. However, we did not find significative alteration in the TNF-α and IL-6 levels of the group treated with isoorientin. It is hard to explain why the effect of isoorientin on the IL-1β levels was more prominent. One could suggest that a differential effect on the NOD-like Receptor Pyrin Domain Containing 3 (NLRP3) pathway would be a reasonable explanation for these findings, since this is a pathway that is important for the formation of IL-1β [31]. Some studies have shown that the anti-inflammatory action of isoorientin is related to the inhibition of the NLRP3 pathway in models of lipopolysaccharide (LPS)-induced acute lung injury in rats [32] and trinitrobenzene sulfonic acid (TNBS)-induced colitis in mice [33], although, even in these studies, the effects of isoorientin extend to other pro-inflammatory cytokines. So, it is difficult to explain these facts clearly from a mechanistic perspective. Despite this, we can securely correlate the reduction in MPO activity, along with the decrease in IL-1β, with the anti-inflammatory effect of isoorientin at the wound site, and these results are consistent with the findings from other authors [34], who have demonstrated reduced infiltration of cells linked to the inflammatory process in mice undergoing treatment with isoorientin. Furthermore, the expression of inflammatory mediators, including cyclooxygenase-2, TNF-α, IL-1β, inducible nitric oxide synthase, and 5-lypoxygenase, decreased in response to treatment with isoorientin in a mice macrophage lineage (Raw 264.7), as well as in vivo in carrageenan-induced air pouch leukocyte recruitment and paw edema models [34].

Thus, our data show that isoorientin modulates the inflammatory response by reducing the release of pro-inflammatory cytokines, which helps prevent excessive inflammation that could impair efficient wound healing. Furthermore, in vitro and in vivo studies [17] have shown that this isoflavone exerts potent antioxidant effects, neutralizing reactive oxygen species (ROS) and reducing oxidative stress at the injury site [35], which may take place through the positive modulation of the nuclear factor erythroid 2-related factor 2 (Nrf2) pathway, as proposed by other authors in mice models such as LPS-induced lung injury [32], ovalbumin-induced asthma [36], and TNBS-induced colitis [33]. A more recent study [37] has also shown that ISO can prevent redox damage to RAW264.7 cells, stimulated by the receptor activation of nuclear factor-κB ligand (RANKL), through a mechanism related to Nrf2. This action may also be crucial for the healing process, as excessive formation of ROS can cause additional tissue damage and delay repair [38].

## 4. Materials and Methods

### 4.1. Groups and Treatments

All procedures were conducted according to the guidelines of the National Council for Control of Animal Experimentation and approved by the Ethics Committee on Animal Use of the Federal University of Sergipe (protocol number 4639261223).

Male Swiss mice (25–30 g) were obtained from the Animal Center of the Federal University of Sergipe. All animals had similar baseline characteristics and were housed in appropriate boxes (dimensions 29 × 18 × 16 cm), with a maximum of 6 animals per box. The distribution of the boxes in the room was aleatory and the mice were kept under standard conditions (22 ± 1 °C, humidity 60 ± 5%, and 12/12 h light/dark cycle) and fed standard laboratory chow and tap water ad libitum.

The animals were individually housed and acclimatized for 7 days before wound induction. We randomly allocated the animals into three experimental groups: control, vehicle, and 2.5% Iso. For the treatment, isoorientin (ChemFaces, Wuhan, China, 98% purity), at a concentration of 2.5%, was applied as a volume of 30 µL (750 µg of the compound) dissolved in DMSO (2%) and propylene glycol (98%) solution. This concentration of isoorientin was based on a previous study [6]. The vehicle was chosen after preliminary tests, considering the poor water solubility of isoorientin. Thus, the vehicle group was treated with 2% DMSO in propylene glycol. The control group did not receive any treatment.

The animals were euthanized 3 (n = 10), 7 (n = 10), or 14 (n = 6) experimental days after the induction (Figure 1A). Experimental procedures were performed in the afternoon.

### 4.2. Wound Healing Model

We selected a mouse model of excisional wounds because it is one of the most used models and it is considered to mimic acute clinical wounds that heal by second intention [39]. This model has been extensively used in prior studies, providing a robust framework for understanding the mechanisms involved in wound healing [19].

For wound induction, mice were anesthetized with ketamine (100 mg/kg, i.p.) and xylazine (10 mg/kg, i.p.). Trichotomy of the dorsothoracic region was performed, followed by antisepsis with 0.5% alcoholic chlorhexidine. Subsequently, cutaneous excision was induced with a 6 mm circular metallic punch. Treatment and vehicle were applied (30 µL) daily from day 0 to day 13 (Figure 1A). On the 14th day, the wound collection was performed with an 8 mm circular metallic punch, encompassing the entire area of the lesion and some healthy tissue, as previously described [40].

The cranio-caudal and latero-lateral diameters of the wounds were measured using a digital caliper on days 0, 3, 7, and 14 (Figure 1A). The wound area was calculated according to the following equation: Wound area (mm^2^) = π.R.r (π = 3.1416), where R = cranio-caudal radius, and r = latero-lateral radius [41]. The researcher responsible for handling the treatments and measurement was not aware of the identity of each group.

### 4.3. Histopathology

The wounds (8 mm diameter) were collected 14 days into the experiment and fixed in 10% formalin for subsequent routine staining with hematoxylin and eosin or Masson’s trichrome. In these experiments, we added a naïve control group (healthy skin) for comparison.

Histological evaluation of the average epidermal tissue thickness was performed on sections stained with hematoxylin and eosin. Three histological fields (100×) in each section were analyzed, and the measurements of epidermal thickness (stratified squamous epithelium lining, excluding the superficial keratin layer) were taken from each field. Morphometric measurements were obtained using ImageJ software (version 1.44).

The analysis of Dermal Collagen Optical Density (DCOD) OCD was performed using ImageJ software (version 1.44). Initially, a photomicrograph (400×) of the tissues stained with Masson’s trichrome was transformed into an 8-bit grayscale system. Next, the software was calibrated using a color palette ranging from white to black. The optical density (OD) values obtained were matched to the corresponding values provided by the software system (standard) through a fitted curve equation (R^2^ = 0.9924), used to calculate collagen OD based on its equivalence to previously calibrated grayscale values. After adjusting the software calibration for the micrometer, optical densities were measured within a circumference of 0.1256 mm^2^ in the papillary/reticular dermis region. For each photomicrograph (100×), OD measurement was carried out in the central area. We performed the measurements in three histological fields in each section of each animal. The pathologist responsible for the measurement was blinded to the groups’ identities.

### 4.4. MPO Activity

Neutrophil infiltration in wounds was indirectly measured by assaying MPO activity as a quantitative marker of the activity of this cell type [42]. Briefly, tissue samples (n = 5 mice) were homogenized in sodium phosphate buffer and 0.5% hexadecyltrimethylammonium bromide (Merck, Darmstadt, Germany), 1 mL/100 mg of tissue. Subsequently, the samples were centrifuged for 2 min at 8000× *g*. The supernatant (20 µL) was added to 200 µL of O-dianisidine (Merck, Darmstadt, Germany) solution, and each sample’s absorbance was determined at 460 nm over 2 min at 25 °C on a plate spectrophotometer (SynergyMx^®^, Biotek, Bad Friedrichshall, Germany).

### 4.5. Determination of Cytokine Concentrations

The samples from 5 mice were homogenized in cytokine extraction buffer containing phosphate-buffered saline (10 mM, pH 7.4), a cocktail of protease inhibitors (1:1000 *v*/*v*; Merck, Darmstadt, Germany), phenylmethylsulfonyl fluoride (0.1 mM), NaCl (150 mM), EDTA (1 mM), and Tween 20 (0.05%), and centrifuged at 10,000× *g*. Subsequently, TNF-α, IL-1β, and IL-6 concentrations in the supernatant were determined using commercially available enzyme-linked immunosorbent assay kits, following the manufacturer’s protocol (Invitrogen, Waltham, MA, USA).

### 4.6. Statistical Analysis

GraphPad Prism 8.0 was utilized for statistical analysis. The Shapiro–Wilk normality test was applied to the data. Data from the wound area (Figure 1B) showed parametric distribution, and two-way ANOVA, followed by the Bonferroni post hoc test, was used. Data regarding the MPO activity and cytokine levels (Figure 4) were also parametric, and a t-test was used. Data concerning the epidermal thickness (Figure 2M) and collagen density (Figure 3I) did not show parametric distribution, and the Kruskal–Wallis test, followed by Dunn’s post hoc test, was used. Data were expressed as the mean ± SD or the median and interquartile range. Values of *p* < 0.05 were considered statistically significant.

## 5. Conclusions

Our data show that isoorientin can improve the healing of excised skin by producing an anti-inflammatory effect in the initial stages of the wound healing process, which results in better epidermal thickness and collagen deposition in the closed wound. These findings reinforce the therapeutic value of isoorientin as an anti-inflammatory agent and an important modulator of fundamental cellular processes for wound healing.

## Data Availability

Data are contained within the article.

## References

[1] Sen C.K. (2021). Human Wound and Its Burden: Updated 2020 Compendium of Estimates. Adv. Wound Care.

[2] Do H.T.T., Edwards H., Finlayson K. (2016). Identifying Relationships between Symptom Clusters and Quality of Life in Adults with Chronic Mixed Venous and Arterial Leg Ulcers. Int. Wound J..

[3] Wilkinson H.N., Hardman M.J. (2020). Wound Healing: Cellular Mechanisms and Pathological Outcomes. Open Biol..

[4] Sorg H., Sorg C.G.G. (2023). Skin Wound Healing: Of Players, Patterns, and Processes. Eur. Surg. Res..

[5] Shaydakov M.E., Ting W., Sadek M., Aziz F., Diaz J.A., Raffetto J.D., Marston W.A., Lal B.K., Welch H.J., Shaydakov M. (2022). Review of the Current Evidence for Topical Treatment for Venous Leg Ulcers. J. Vasc. Surg. Venous Lymphat. Disord..

[6] Duque A.P.d.N., Pinto N.d.C.C., Mendes R.d.F., da Silva J.M., Aragão D.M.d.O., Castañon M.C.M.N., Scio E. (2016). In Vivo Wound Healing Activity of Gels Containing Cecropia Pachystachya Leaves. J. Pharm. Pharmacol..

[7] Okonkwo U.A., DiPietro L.A. (2017). Diabetes and Wound Angiogenesis. Int. J. Mol. Sci..

[8] Rosen J., Landriscina A., Kutner A., Adler B.L., Krausz A.E., Nosanchuk J.D., Friedman A.J. (2015). Silver Sulfadiazine Retards Wound Healing in Mice via Alterations in Cytokine Expression. J. Investig. Dermatol..

[9] Wang X., Izzo A.A., Papapetropoulos A., Alexander S.P.H., Cortese-Krott M., Kendall D.A., Martemyanov K.A., Mauro C., Panettieri R.A., Patel H.H. (2024). Natural Product Pharmacology: The British Journal of Pharmacology Perspective. Br. J. Pharmacol..

[10] Harvey A.L., Edrada-Ebel R., Quinn R.J. (2015). The Re-Emergence of Natural Products for Drug Discovery in the Genomics Era. Nat. Rev. Drug Discov..

[11] Al-Khayri J.M., Sahana G.R., Nagella P., Joseph B.V., Alessa F.M., Al-Mssallem M.Q. (2022). Flavonoids as Potential Anti-Inflammatory Molecules: A Review. Molecules.

[12] Choy K.W., Murugan D., Leong X.-F., Abas R., Alias A., Mustafa M.R. (2019). Flavonoids as Natural Anti-Inflammatory Agents Targeting Nuclear Factor-Kappa B (NFκB) Signaling in Cardiovascular Diseases: A Mini Review. Front. Pharmacol..

[13] Sychrová A., Škovranová G., Čulenová M., Fialová S.B. (2022). Prenylated Flavonoids in Topical Infections and Wound Healing. Molecules.

[14] González R., Ballester I., López-Posadas R., Suárez M.D., Zarzuelo A., Martínez-Augustin O., Medina F.S.D. (2011). Effects of Flavonoids and Other Polyphenols on Inflammation. Crit. Rev. Food Sci. Nutr..

[15] Fan X., Wei W., Huang J., Liu X., Ci X. (2020). Isoorientin Attenuates Cisplatin-Induced Nephrotoxicity Through the Inhibition of Oxidative Stress and Apoptosis via Activating the SIRT1/SIRT6/Nrf-2 Pathway. Front. Pharmacol..

[16] Yuan L., Wu Y., Ren X., Liu Q., Wang J., Liu X. (2014). Isoorientin Attenuates Lipopolysaccharide-Induced pro-Inflammatory Responses through down-Regulation of ROS-Related MAPK/NF-κB Signaling Pathway in BV-2 Microglia. Mol. Cell Biochem..

[17] Yuan L., Han X., Li W., Ren D., Yang X. (2016). Isoorientin Prevents Hyperlipidemia and Liver Injury by Regulating Lipid Metabolism, Antioxidant Capability, and Inflammatory Cytokine Release in High-Fructose-Fed Mice. J. Agric. Food Chem..

[18] Wedler J., Daubitz T., Schlotterbeck G., Butterweck V. (2014). In Vitro Anti-Inflammatory and Wound-Healing Potential of a Phyllostachys Edulis Leaf Extract–Identification of Isoorientin as an Active Compound. Planta Med..

[19] Sami D.G., Heiba H.H., Abdellatif A. (2019). Wound Healing Models: A Systematic Review of Animal and Non-Animal Models. Wound Med..

[20] Criollo-Mendoza M.S., Contreras-Angulo L.A., Leyva-López N., Gutiérrez-Grijalva E.P., Jiménez-Ortega L.A., Heredia J.B. (2023). Wound Healing Properties of Natural Products: Mechanisms of Action. Molecules.

[21] Ziqubu K., Dludla P.V., Joubert E., Muller C.J.F., Louw J., Tiano L., Nkambule B.B., Kappo A.P., Mazibuko-Mbeje S.E. (2020). Isoorientin: A Dietary Flavone with the Potential to Ameliorate Diverse Metabolic Complications. Pharmacol. Res..

[22] Peña O.A., Martin P. (2024). Cellular and Molecular Mechanisms of Skin Wound Healing. Nat. Rev. Mol. Cell Biol..

[23] Altoé L.S., Alves R.S., Sarandy M.M., Morais-Santos M., Novaes R.D., Gonçalves R.V. (2019). Does Antibiotic Use Accelerate or Retard Cutaneous Repair? A Systematic Review in Animal Models. PLoS ONE.

[24] Ebaid H. (2014). Neutrophil Depletion in the Early Inflammatory Phase Delayed Cutaneous Wound Healing in Older Rats: Improvements Due to the Use of Un-Denatured Camel Whey Protein. Diagn. Pathol..

[25] Hassanshahi A., Moradzad M., Ghalamkari S., Fadaei M., Cowin A.J., Hassanshahi M. (2022). Macrophage-Mediated Inflammation in Skin Wound Healing. Cells.

[26] Boniakowski A.E., Kimball A.S., Jacobs B.N., Kunkel S.L., Gallagher K.A. (2017). Macrophage-Mediated Inflammation in Normal and Diabetic Wound Healing. J. Immunol..

[27] Antunes-Ricardo M., Gutiérrez-Uribe J., Serna-Saldívar S.O. (2015). Anti-Inflammatory Glycosylated Flavonoids as Therapeutic Agents for Treatment of Diabetes-Impaired Wounds. Curr. Top. Med. Chem..

[28] Kant V., Jangir B.L., Sharma M., Kumar V., Joshi V.G. (2021). Topical Application of Quercetin Improves Wound Repair and Regeneration in Diabetic Rats. Immunopharmacol. Immunotoxicol..

[29] Lodhi S., Singhai A.K. (2013). Wound Healing Effect of Flavonoid Rich Fraction and Luteolin Isolated from *Martynia annua* Linn. on Streptozotocin Induced Diabetic Rats. Asian Pac. J. Trop. Med..

[30] Landén N.X., Li D., Ståhle M. (2016). Transition from Inflammation to Proliferation: A Critical Step during Wound Healing. Cell. Mol. Life Sci..

[31] Ding Y., Ding X., Zhang H., Li S., Yang P., Tan Q. (2022). Relevance of NLRP3 Inflammasome-Related Pathways in the Pathology of Diabetic Wound Healing and Possible Therapeutic Targets. Oxid. Med. Cell Longev..

[32] Zhang L., Zhu X.-Z., Badamjav R., Zhang J.-Z., Kou J.-P., Yu B.-Y., Li F. (2022). Isoorientin Protects Lipopolysaccharide-Induced Acute Lung Injury in Mice via Modulating Keap1/Nrf2-HO-1 and NLRP3 Inflammasome Pathways. Eur. J. Pharmacol..

[33] Cheng Q., Yu X., Zhang R., Chen L. (2020). Isoorientin Alleviates Inflammatory Bowel Disease by Inhibiting NLRP3 Inflammasome Activation through Nrf2/NQO1 Pathway. Curr. Top. Nutraceutical Res..

[34] Anilkumar K., Reddy G.V., Azad R., Yarla N.S., Dharmapuri G., Srivastava A., Kamal M.A., Pallu R. (2017). Evaluation of Anti-Inflammatory Properties of Isoorientin Isolated from Tubers of *Pueraria tuberosa*. Oxid. Med. Cell Longev..

[35] Zhao Y., He C., Hu S., Ni H., Tan X., Zhi Y., Yi L., Na R., Li Y., Du Q. (2024). Anti-Oxidative Stress and Cognitive Improvement of a Semi-Synthetic Isoorientin-Based GSK-3β Inhibitor in Rat Pheochromocytoma Cell PC12 and Scopolamine-Induced AD Model Mice via AKT/GSK-3β/Nrf2 Pathway. Exp. Neurol..

[36] Liang S., Zhao Y., Chen G., Wang C. (2022). Isoorientin Ameliorates OVA-Induced Asthma in a Murine Model of Asthma. Exp. Biol. Med..

[37] Zhang B., Qu Z., Hui H., He B., Wang D., Zhang Y., Zhao Y., Zhang J., Yan L. (2024). Exploring the Therapeutic Potential of Isoorientin in the Treatment of Osteoporosis: A Study Using Network Pharmacology and Experimental Validation. Mol. Med..

[38] Dunnill C., Patton T., Brennan J., Barrett J., Dryden M., Cooke J., Leaper D., Georgopoulos N.T. (2017). Reactive Oxygen Species (ROS) and Wound Healing: The Functional Role of ROS and Emerging ROS-Modulating Technologies for Augmentation of the Healing Process. Int. Wound J..

[39] Masson-Meyers D.S., Andrade T.A.M., Caetano G.F., Guimaraes F.R., Leite M.N., Leite S.N., Frade M.A.C. (2020). Experimental Models and Methods for Cutaneous Wound Healing Assessment. Int. J. Exp. Pathol..

[40] Canesso M.C.C., Vieira A.T., Castro T.B.R., Schirmer B.G.A., Cisalpino D., Martins F.S., Rachid M.A., Nicoli J.R., Teixeira M.M., Barcelos L.S. (2014). Skin Wound Healing Is Accelerated and Scarless in the Absence of Commensal Microbiota. J. Immunol..

[41] Ramsey D.T., Pope E.R., Wagner-Mann C., Berg J.N., Swaim S.F. (1995). Effects of Three Occlusive Dressing Materials on Healing of Full-Thickness Skin Wounds in Dogs. Am. J. Vet. Res..

[42] Bradley P.P., Priebat D.A., Christensen R.D., Rothstein G. (1982). Measurement of Cutaneous Inflammation: Estimation of Neutrophil Content with an Enzyme Marker. J. Investig. Dermatol..

