# Correction: Hora et al. Isoorientin Improves Excisional Skin Wound Healing in Mice. *Pharmaceuticals* 2024, *17*, 1368

**DOI:** 10.3390/ph18091343

**Published:** 2025-09-08

**Authors:** Aline B. Hora, Laiza S. Biano, Ana Carla S. Nascimento, Zaine T. Camargo, Greice I. Heiden, Ricardo L. C. Albulquerque-Júnior, Renata Grespan, Jessica M. D. A. Aragão, Enilton A. Camargo

**Affiliations:** 1 Graduate Program in Health Sciences, Federal University of Sergipe, São Cristóvão 49060-676, Brazil; 2 Graduate Program in Physiological Sciences, Federal University of Sergipe, São Cristóvão 49107-230, Brazil; 3 Graduate Program in Chemistry, Federal University of Sergipe, São Cristóvão 49107-230, Brazil; 4 Graduate Program in Dentistry, Federal University of Santa Catarina, Florianópolis 88040-900, Brazil

In the original publication [1], there were mistakes in affiliation for author Renata Grespan and Figure 2 as published. 

The correct affiliation for Renata Grespan is 2. 

An error was made during the assembly of the figure, resulting in the inadvertent duplication of the same image for two different treatment conditions. The written description of the results in the manuscript is accurate, correctly reflecting the distinct morphological features observed in each treatment group. The error is restricted to the photomicrographic panel, where an incorrect image was mistakenly included, which does not correspond to the text. The corrected Figure 2 appears below. The authors state that the scientific conclusions are unaffected. This correction was approved by the Academic Editor. The original publication has also been updated.

**Figure 2:**
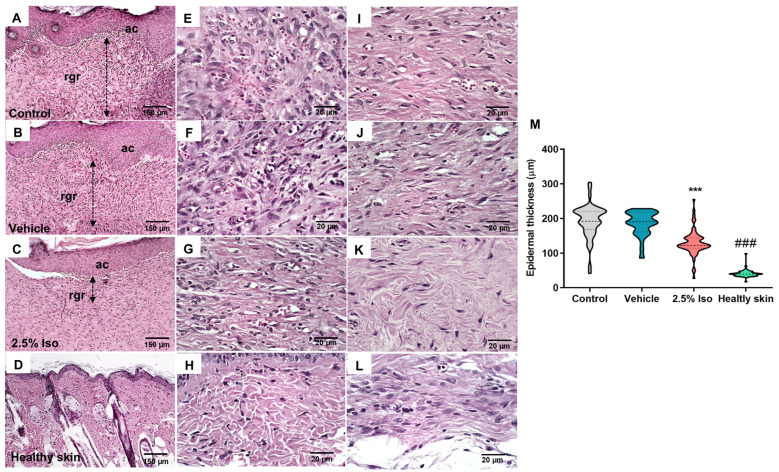
Representative images of the wound tissues 14 days after induction and quantification of the epidermal thickness. Histological sections stained with hematoxylin and eosin represent each experimental group’s epidermis and papillary/reticular dermis. Panoramic views of the groups—control (**A**), vehicle (**B**), 2.5% Iso (**C**), and healthy skin (**D**)—show the measurement of the granulation reaction depth (dotted lines with double arrows), epidermal acanthosis (ac), and residual granulation reaction (rgr) in the lamina propria (100×). Details of the granulation reaction and the deeper dermal portion in the control (**E**,**I**), vehicle (**F**,**J**), 2.5% Iso (**G**,**K**), and healthy skin (**H**,**L**) groups (400×) are shown. A violin plot shows the measurement of the mean epidermal thickness in the experimental groups (**M**), n = 6 animals with 6–8 measurements each. The Kruskal–Wallis and Dunn’s post hoc tests were used. *** *p* < 0.001 and ^###^ *p* < 0.001 compared to the control or vehicle groups.
